# Accessible water quality monitoring through hybrid human–machine colorimetric methods

**DOI:** 10.1007/s10661-025-13983-x

**Published:** 2025-04-15

**Authors:** Dakota Aaron McCarty, Minji Alyssa Kim, Hyunwoo Jo, Eunchong Yim, Hayoung Yun, Samuel Sims, Minji Kim, Soyoung Kwon

**Affiliations:** 1 Department of Environmental Science and Policy, George Mason University - Korea, Incheon, South Korea; 2 Department of Global Affairs, George Mason University - Korea, Incheon, South Korea; 3 Center for Security Policy Studies - Korea, Incheon, South Korea

**Keywords:** Water quality assessment, Colorimetric test strips, RGB analysis, Human–machine methods, Environmental monitoring

## Abstract

Effective water quality monitoring is important for environmental protection and public health, yet conventional field and laboratory methods each present significant limitations. Field tools such as colorimetric test strips offer affordability and accessibility but are prone to subjective interpretation and environmental variability. In contrast, laboratory-based techniques provide high precision but are costly, resource-intensive, and less feasible in decentralized contexts. This study presents a hybrid human–machine methodology that improves the accuracy and reproducibility of colorimetric test strip analysis while maintaining field-level accessibility. A total of 34 water samples collected along a 7-km stretch of Seunggi Stream in Incheon, South Korea, were analyzed using a web-based platform that extracts RGB values from images of test strips and reference charts. To translate color into concentration, the system calculates Euclidean distances between test strip colors and known reference values, then applies inverse distance weighting (IDW) to interpolate continuous estimates from the closest matches. This approach overcomes the limitations of discrete reference charts, enabling more precise and reproducible readings without the need for complex machine learning models. Validation against standard laboratory methods revealed strong correlations (*r* > 0.85 for pH, lead, and total hardness), supporting the reliability of the approach. Spatial trends in pollutants were successfully mapped, demonstrating the method’s utility for environmental monitoring. This cost-effective, scalable solution bridges the gap between subjective field testing and laboratory precision, offering a practical tool for resource-limited settings, citizen science, and preliminary assessments. Future research will refine analyte-specific accuracy and expand applicability to more diverse conditions.

## Introduction

Water quality monitoring is essential for maintaining environmental and public health, ecological integrity, and for sustainable water management. Despite its importance, water quality often receives less attention compared to other issues related to water availability, even as pollutants from anthropogenic activities increasingly threaten freshwater systems (Häder et al., 2020; Rhind, 2009; World Health Organization et al., 2022). These contaminants, ranging from nitrates and heavy metals to complex organic compounds, compromise the health of aquatic ecosystems and present significant risks to human well-being (Chapman & Sullivan, 2022; Rhind, 2009). Researchers have found that a quarter of the global population still lacks access to a reliable source of safe drinking water, while one-third lacks adequate sanitation facilities (Swinney et al., 2024; Thomas, 2024). This disparity disproportionately impacts low-income communities and impoverished urban areas worldwide (Häder et al., 2020; Swanston et al., 1994). Contaminated water remains a major factor contributing to reduced life expectancy in these regions (Mújica et al., 2015).

Methods for water quality assessment range from inexpensive field tools to advanced laboratory technologies. At one end of the spectrum are colorimetric test strips, which provide a practical and cost-effective means for detecting chemical parameters such as pH, nitrates, and heavy metals in water samples (Waters et al., 1995). These tools are widely used due to their affordability and portability, making them particularly valuable in resource-limited settings, in citizen science initiatives, but also with hobby environmentalists (D’Alessio et al., 2021; Malina et al., 2024; Ruppen et al., 2021; Sicard et al., 2015; Waters et al., 1995). Test strips require minimal training, can be easily transported to field sites, and allow for rapid water quality checks without the need for advanced laboratory infrastructure, holding the potential to empower a broader range of users to participate in environmental monitoring efforts. However, the reliance on visual interpretation to compare test strip color changes against standardized charts potentially introduces large amounts variability, as outcomes can be influenced by factors such as lighting, user expertise, and individual differences in color perception (Kılıç et al., 2018; Morbioli et al., 2017; Muhammad-Aree & Teepoo, 2020; Zwinkels, 1997).

Conversely, laboratory-based methods such as spectrophotometry, high-performance liquid chromatography (HPLC), and titration provide highly accurate and precise measurements. These techniques, while reliable, are costly, require specialized equipment and materials, and are not readily accessible for routine or widespread application, particularly in regions with limited resources (Pereira & Hosker, 2019; Sicard et al., 2015). The gap between accessible yet variable field methods and resource-intensive laboratory techniques underscores the need for new approaches to water quality monitoring (Chapman & Sullivan, 2022).

Advancements in digital imaging and computational analysis have introduced new possibilities for enhancing the reliability of colorimetric test strips. Smartphone-based systems, for example, use built-in cameras to capture test strip images and employ algorithms to analyze color changes. Such methods have shown promise in reducing subjectivity and improving reproducibility (Flaucher et al., 2022; Kılıç et al., 2018; Muhammad-Aree & Teepoo, 2020). However, these systems still face challenges related to lighting variability, image quality, and the limitations of traditional reference charts, which often provide only discrete concentration values, leading to loss of precision (Martinez et al., 2008; Morbioli et al., 2017).

Recent studies have proposed enhancements to mitigate these limitations. For example, Flaucher et al. (2022) incorporated smartphone-based prenatal care test strip analysis, demonstrating the utility of color correction and signal averaging for more consistent results. Similarly, Swinney et al. (2024) found that multi-test strips used in at-home water testing can achieve reasonably high precision when paired with consistent lighting and standardized reading conditions. Approaches that leverage gray or white reference cards for color calibration are gaining traction as a way to further reduce color perception variability in uncontrolled environments (Sicard et al., 2015).

In parallel, machine learning and deep learning methods have been increasingly adopted to enhance colorimetric interpretation. Deep convolutional neural networks (CNNs), such as those employed by Zhang et al. (2024), enable highly accurate parameter detection across multiple analytes in water samples under varied lighting conditions. Some frameworks have incorporated object detection and classification tasks, allowing the simultaneous identification of multiple test strip zones with automated concentration estimation. These approaches significantly improve performance but often require extensive training datasets, high-resolution images, and computational resources, making them difficult to implement in decentralized or resource-limited contexts (Morbioli et al., 2017; Zhang et al., 2024).

Additionally, other work has explored the integration of paper-based microfluidic devices with smartphone imaging (Martinez et al., 2008; Morbioli et al., 2017) and the use of Bluetooth-enabled digital readers or cloud-based platforms for real-time water quality reporting (Sicard et al., 2015). These developments point toward a future where distributed, digital water monitoring can complement centralized laboratory analyses and expand access to timely environmental data.

Despite these advances, the need remains for methods that balance accessibility, precision, and ease of use. The methodology presented in this study builds on these developments by offering a low-resource alternative that enhances the accuracy of colorimetric test strip analysis without requiring complex infrastructure or machine learning expertise. This study addresses these challenges by introducing a hybrid human–machine methodology for interpreting colorimetric test strip results. This approach utilizes a web-based platform to extract Red–Green–Blue (RGB) color values from test strips and reference charts within captured images. Euclidean distance calculations are applied to match test strip colors with the closest reference values, and inverse distance weighting is used to interpolate between these values, providing continuous concentration estimates. By leveraging computational tools to enhance the interpretation of field-based tests, this methodology aims to bridge the gap between accessibility and precision in water quality monitoring (Kılıç et al., 2018; Sanda Mahama et al., 2016).

This research contributes to the field by providing a scalable and cost-effective method that improves the accuracy and reproducibility of colorimetric analysis. In this context, scalability refers primarily to the deployment feasibility and cost-effectiveness of the method, enabling expansion across different geographic regions or larger monitoring networks without large infrastructure investments. Beyond its application to water quality, the proposed approach holds potential for other fields reliant on colorimetric testing, including environmental monitoring and diagnostic assays. By addressing the limitations of traditional visual methods and digital adaptations, this work advances the practical utility of colorimetric test strips for broad environmental and public health applications. That being said, it should be noted that this research does not aim to replace the more accurate lab-based testing, rather, it aims to assist in areas where such methods are not available or where it would make more sense to run preliminary tests with colorimetric methods before going into the lab due to cost or resource limitations.

The proposed methodology of study additionally aligns closely with global initiatives such as the United Nations’Sustainable Development Goals (SDGs), particularly Goal 6: *Clean Water and Sanitation *(Arora & Mishra, 2022). By providing a scalable, cost-effective solution for water quality monitoring, this approach addresses the critical need for accessible tools in resource-limited settings, where the lack of reliable water quality assessments exacerbates public health challenges and environmental degradation. The hybrid human–machine method empowers communities, researchers, and policymakers to monitor water quality more efficiently, thereby contributing to targets such as improving water quality by reducing pollution, minimizing the release of hazardous chemicals, and supporting capacity-building initiatives for water management. Through its emphasis on inclusivity and precision, this approach has the potential to accelerate progress toward ensuring universal access to safe and sustainable water resources, bridging gaps in existing monitoring frameworks.

## Materials and methods

### Study area and sample collection

**Fig. 1:**
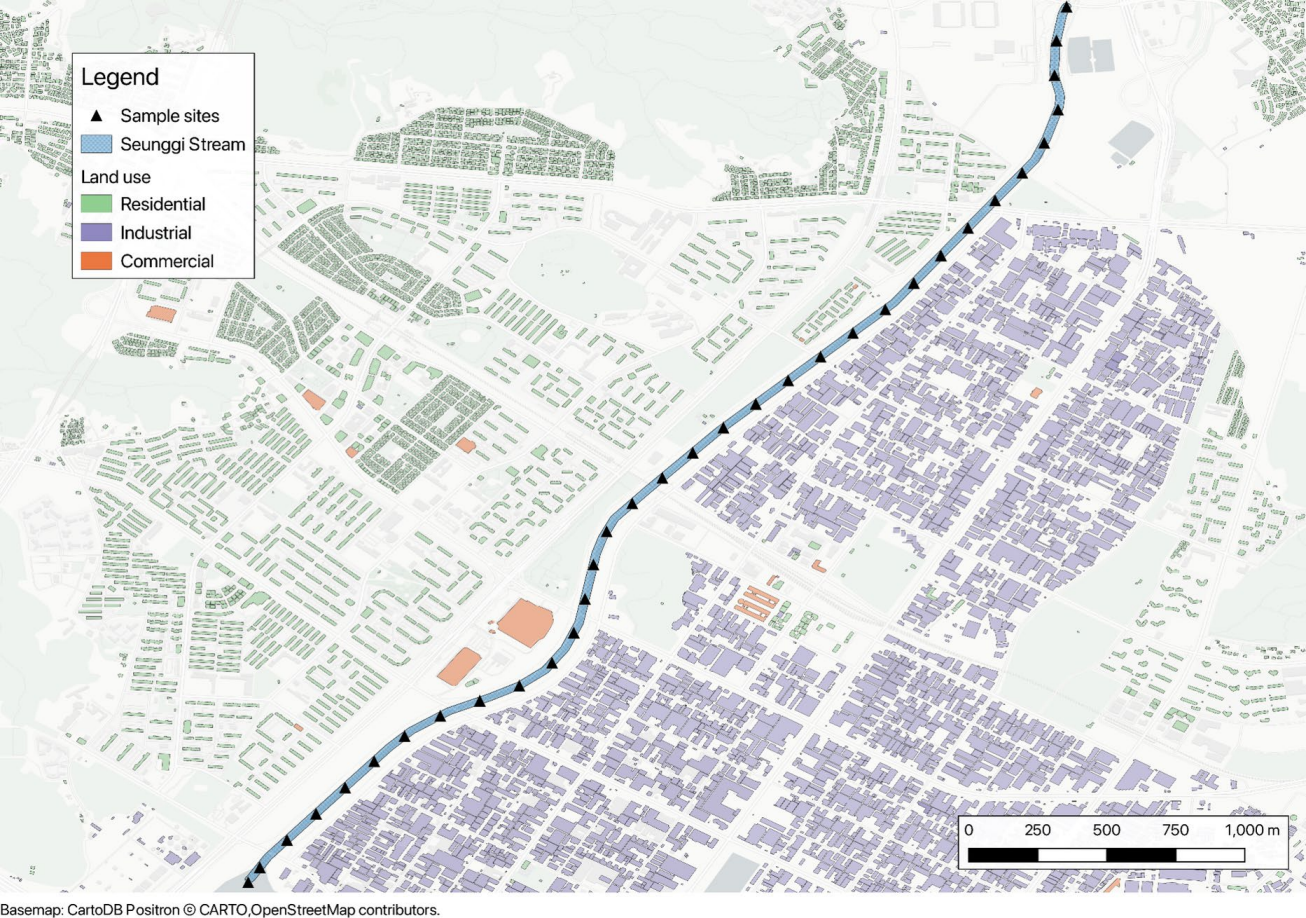
Map of Seunggi Stream, sample sites, and surrounding land use

To evaluate the effectiveness of the proposed colorimetric interpretation method, a field study was conducted along the Seunggi Stream in Incheon, South Korea (Fig. 1). A total of 34 water samples were collected at approximately 200-m intervals, covering a 7-km stretch of the stream to capture spatial variations in water quality parameters. At each sampling site, water samples were collected following standard protocols to ensure consistency and prevent contamination. Samples were stored in coolers and later analyzed off-site.

Seunggi Stream has been the site of regular criticism from the public and environmental activists which has led to multiple government funded projects to improve the water quality in the stream (K.-S. Lee et al., 2014; S. Lee & Choi, 2012; Seo, 2001; Sin & Jo, 2001). These aspects were a main consideration in the site selection as this is an ideal location that could benefit from enhanced water quality monitoring methods. The proposed approach has the potential to empower citizens, organizations, and municipal authorities to monitor the stream more regularly, tracking the effectiveness of interventions without relying solely on advanced and expensive laboratory testing.

The stream is bordered by residential areas on its western banks and an industrial complex to the east. Additionally, a large commercial center, Square One, is situated along a bend in the stream. Although riparian zones and other protective measures have been implemented (Sin & Jo, 2001), the water quality of the stream remains below environmental standards (S. Lee & Choi, 2012). While the introduction of an improved methodology for water quality testing would not directly enhance the stream’s condition, it could empower citizens to conduct more accessible and frequent assessments. This, in turn, would facilitate greater accountability for local government and relevant organizations, while also enabling continuous monitoring of efforts to improve stream quality.

### Water quality test strips

This study uses 16-in-one multi-test test strips manufactured by Pamasana and Tespert (Bachar et al., 2024; Swinney et al., 2024). These commercially available water quality test strips are capable of measuring parameters such as pH, free chlorine, total hardness, heavy metals, and other relevant indicators. While they are more typically associated with drinking water testing, they provide the most accessible option for colorimetric testing strips due to their affordability and ease of purchasing. After exposure to the water samples, the test strips developed color changes corresponding to the concentrations of the analytes. The test strips were used according to the manufacturer’s instructions.

### Image acquisition and color sampling

Human input played a crucial role in this method by guiding the selection of representative RGB values from both the test strip and the reference chart. This manual step enhanced accuracy and reduced variability during subsequent computational analyses. Rather than employing machine learning-based models, our approach utilized Euclidean distance calculations and inverse distance weighting (IDW) interpolation, chosen specifically for their simplicity, interpretability, and minimal calibration requirements, ensuring greater ease of use and broader accessibility.

**Fig. 2:**
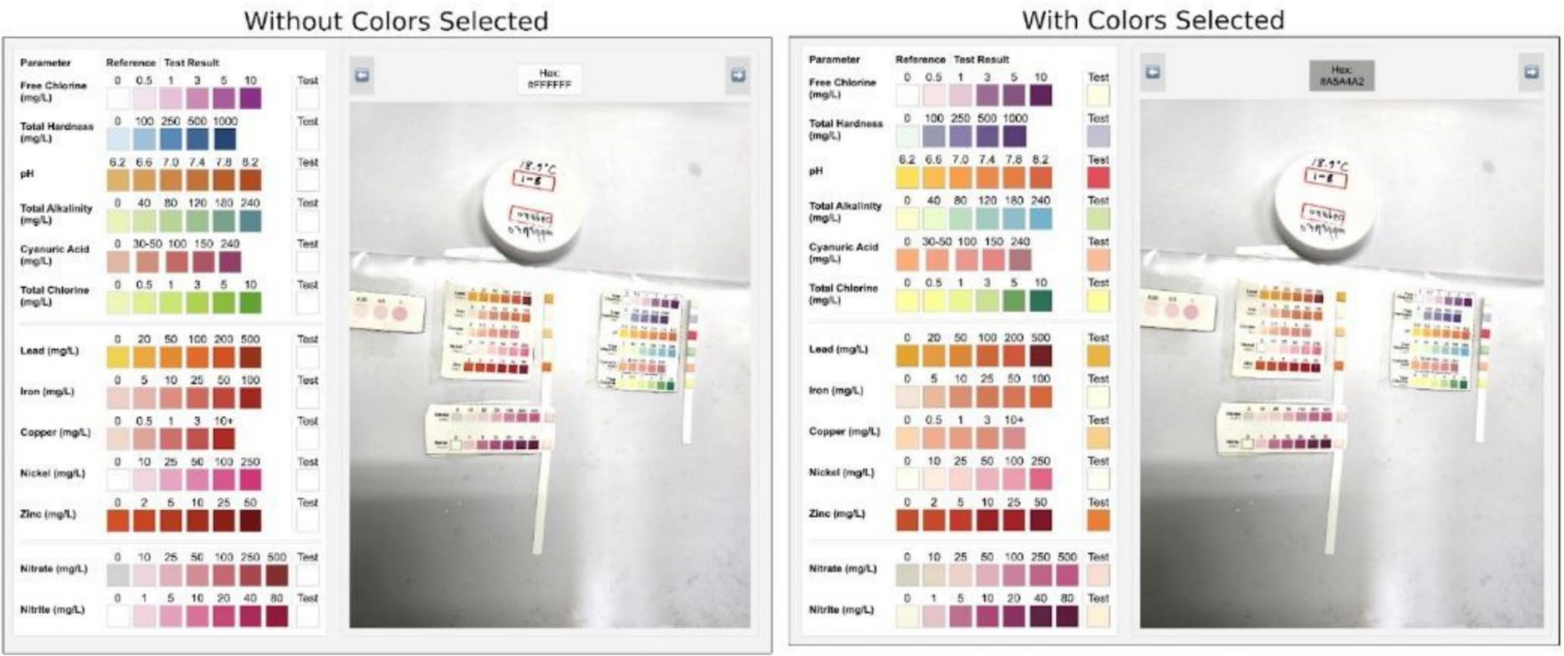
Examples of the color selector UI before and after the reference colors are selected

High-resolution images of the test strips were captured using digital cameras under controlled lighting conditions to minimize variability due to differences in illumination, camera settings, and viewing angles, as these factors impact perceived colors (Morbioli et al., 2017; Sicard et al., 2015). Each test strip was placed alongside a standardized reference parameter sheet, ensuring direct and consistent comparison between test colors and known reference concentrations. During preliminary evaluations, multiple smartphone cameras and intentionally varied conditions (including mild shadowing as illustrated in Fig. 2) were tested to assess method robustness. However, care was consistently taken to ensure identical lighting conditions for both the test strip and the reference chart within each image. For further improvements in accuracy and reproducibility, future studies may incorporate standardized gray or white reference cards for camera calibration and white balancing prior to image capture. However, this additional step could increase procedural complexity and may somewhat reduce the accessibility advantages inherent to the proposed method.

A web-based application (Fig. 2) was developed to facilitate accurate color sampling from the images that can be done by a researcher. This application allowed users to sample RGB values directly from both the test strips (after water exposure and appropriate wait times) and the reference parameter sheet that was provided by the manufacturer, within the same image to limit previously mentioned externalities. This produces a dataset containing:

- Test RGB values: Colors sampled from the test strip for each parameter stored as numerical tuples using “(*R*, *G*, *B*)” as the format.
- Reference RGB values: Colors from the reference parameter sheet, each representing known concentration levels, stored as a dictionary of RGB tuples that can later be referenced to match the test strip against.

The color selection tool provided the option to average the RGB values over 1, 3, or 5 neighboring pixels, allowing users to select the most representative color values and reduce noise due to pixel-level variations. In this method, the aim is to mathematically store the RGB values for both the test strips and the reference sheets to eliminate the need to represent the actual color.

Comparing the “Without Colors Selected” and “With Colors Selected” sections in Fig. 2 highlights how variations in lighting can alter perceived colors, as seen with the “Total Hardness” parameter, which appears slightly more purple after color sampling. The aim of this method is to bypass these perceptual inconsistencies by mathematically encoding color values. This approach shifts the focus of parameter assessment from subjective visual interpretation to an objective analysis of mathematical proximity, minimizing reliance on human color perception.

While manual color selection partially mitigates lighting variability by comparing test and reference regions within the same image, maintaining consistent lighting conditions, avoiding overlapping shadows, etc. remains important to accurate RGB extraction. Although preliminary evaluations indicated robustness across multiple devices, systematic cross-device consistency assessments could be a topic further explored in future research.

### Colorimetric data processing

While machine learning methods, including convolutional neural networks (CNNs) and deep learning approaches, offer potential improvements in prediction accuracy, they demand large, labeled training datasets, computational resources, and extensive calibration efforts. Our selected methods (Euclidean distance and IDW interpolation) were thus chosen to maintain simplicity, interpretability, rapid deployment, and accessibility in resource-limited environmental monitoring settings.

Euclidean distance was selected due to its straightforward interpretability when quantifying color differences, which is particularly beneficial in resource-limited field applications. Additionally, IDW interpolation provides a reliable means to interpolate continuous concentration values from discrete reference points, essential when exact reference matches are absent. Alternative methods, including polynomial regression or machine learning techniques, were considered but ultimately deemed unsuitable due to their complexity, need for extensive calibration datasets, and reduced transparency.

#### RGB conversion and color distance calculation

The RGB values obtained from the test strips and reference sheets were processed to translate color information into concentration estimates. RGB values were parsed into numerical tuples (*R*, *G*, *B*). The Euclidean distance between each test RGB value and each reference RGB value was calculated using the formula:

$$D=\sqrt{{({R}_{1}-{R}_{2})}^{2}+{({G}_{1}-{G}_{2})}^{2}+{({B}_{1}-{B}_{2})}^{2}}$$

where (*R*_1_, *G*_1_, *B*_1_) and (*R*_2_, *G*_2_, *B*_2_) represent the RGB values of the test sample and the reference color, respectively. This objective metric reduces the impact of subjective color interpretation and variations due to image capture conditions (Sanda Mahama et al., 2016).

Each test RGB value was matched to the closest reference RGB value based on the minimum Euclidean distance, establishing an initial concentration estimate for each parameter. This stage is more-or-less replicating the process of a human researcher that would be visually matching the reacted test strip to the reference chart.

#### Inverse distance weighting interpolation

To improve the accuracy of the concentration estimates, an interpolation method based on inverse distance weighting (IDW) was applied for test RGB values that did not exactly match any reference color (Sanda Mahama et al., 2016). For each test RGB value, the two nearest reference colors were identified—one representing a higher concentration and the other a lower concentration. Each of these reference colors was assigned a weight inversely proportional to its distance from the test RGB value:

$$V=\frac{{W}_{1}{V}_{1}+{W}_{2}{V}_{2}}{{W}_{1}+{W}_{2}}$$

where *V* is the interpolated concentration, *V*_1_ and *V*_2_ are the concentrations of the two closest reference points, and *W*_1_ and *W*_2_ are the inverse distances to these points, calculated as ${W}_{i}=\frac{1}{{D}_{i}}$. This weighting ensures that the interpolated concentration more accurately reflects the test color’s position relative to known concentration values.

IDW was specifically chosen because it assigns greater significance to reference colors nearest in RGB space, smoothly converting discrete color keys into continuous concentration estimates. Other methods, such as polynomial regression and machine learning-based interpolation methods, were considered but not pursued, as they impose restrictive functional forms or necessitate substantial training data, which are impractical in resource-limited contexts.

**Fig. 3:**
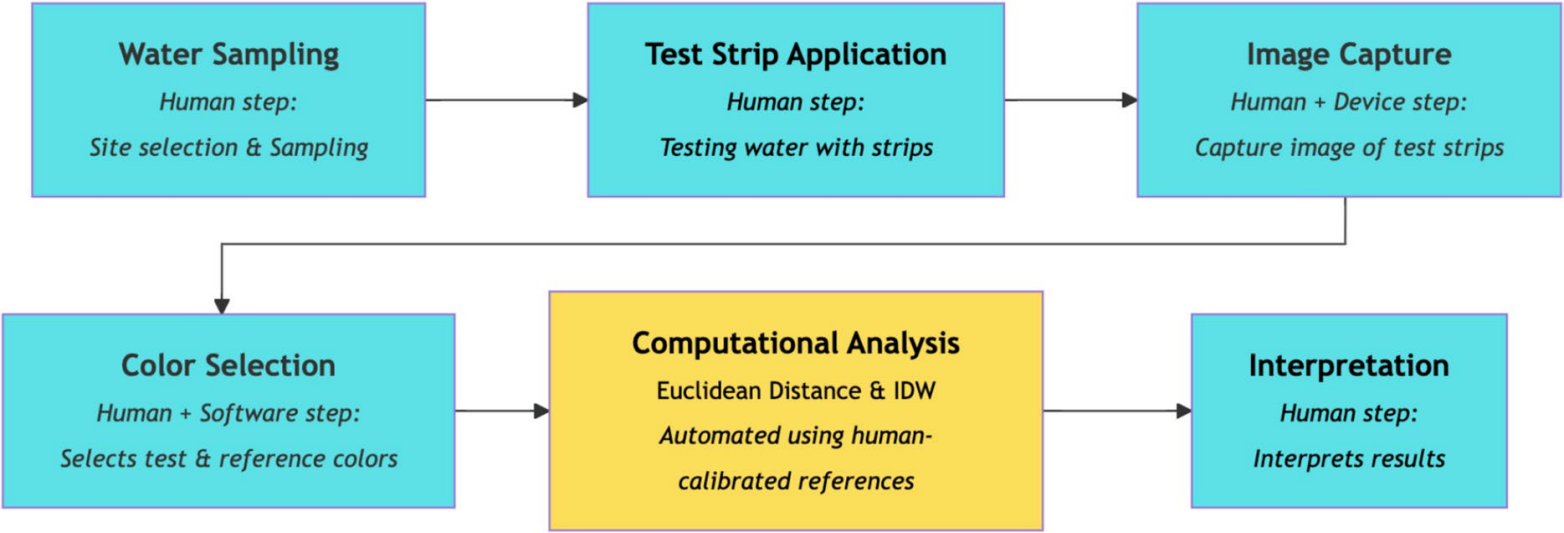
Workflow diagram illustrating the hybrid human–machine colorimetric method for water quality monitoring

Figure 3 below illustrates the process and human–machine interaction workflow in this methodology with blue colors boxes representing human driven steps and the yellow box showing where the Euclidean distance and IDW are used to analyze the colors.

### Laboratory validation

To validate the accuracy and reliability of the proposed colorimetric method, a subset of water samples was analyzed using standard laboratory techniques. Parameters such as pH were measured using a calibrated pH meter according to Standard Methods for the Examination of Water and Wastewater (Apha & Wef, 2017). Free chlorine concentrations were determined through spectrophotometry following the DPD (N,N-diethyl-p-phenylenediamine) method as outlined by the same Standard Methods (Apha & Wef, 2017). Total hardness was assessed using EDTA titration techniques prescribed in these standard protocols (Apha & Wef, 2017). Heavy metals and nutrients were analyzed using atomic absorption spectroscopy (AAS) and ion chromatography (IC) in accordance with established U.S. Environmental Protection Agency (U.S. EPA) methods and Standard Methods for the Examination of Water and Wastewater (Epa, 1994).

### Statistical analysis

The results from the proposed human–machine method, as well as those derived from researcher interpretation of test strips, were compared with laboratory measurements. This comparison aimed not only to evaluate the performance of the suggested methodology against standard laboratory methods but also to assess how it compares to the traditional, human interpretation of test strips. Statistical analyses, including Pearson correlation coefficients (Pearson, 1895) and Bland–Altman plots (Bland & Altman, 2010), were used to quantify the level of agreement between the datasets. These analyses provided insights into the validity of the suggested colorimetric method for water quality assessment, aligning with established practices for evaluating agreement between measurement techniques (Giavarina, 2015).

## Results and discussion

### Correlation validation

Table 1 presents the correlation between the test strip method and laboratory-based approaches for parameter estimation. To evaluate the potential of the new method to enhance estimation accuracy, correlations were analyzed for test strips interpreted by researchers and those assessed using the human–machine approach. In all cases, the results correlation was improved. It should be noted that the process to come to a consensus for some of the parameters was tedious in some cases where the color change on the test strip was not necessarily obvious.

**Table 1:** Correlation coefficients (r), 95% confidence intervals (CI), and p-values comparing visual and human–machine colorimetric test-strip methods to laboratory-based measurements (*n* = 34)

Parameter	Correlation (Visual method)	Correlation (Human–Machine method)
Total Chlorine (mg/L)	0.79*** [0.62–0.89]	0.91*** [0.83–0.95]
Free Chlorine (mg/L)	0.80*** [0.63–0.90]	0.89*** [0.79–0.94]
Iron (mg/L)	0.46** [0.14–0.69]	0.86*** [0.74–0.93]
Total Hardness (mg/L)	0.76*** [0.57–0.87]	0.85*** [0.72–0.92]
Lead (mg/L)	0.48** [0.17–0.70]	0.83*** [0.68–0.91]
pH	0.80*** [0.63–0.90]	0.82*** [0.67–0.91]
Total Alkalinity (mg/L)	0.65*** [0.40–0.81]	0.81*** [0.65–0.90]
Nitrite (mg/L)	0.44** [0.12–0.68]	0.78 *** [0.60–0.88]
Nitrate (mg/L)	0.41** [0.08–0.66]	0.70 *** [0.47–0.84]
Copper (mg/L)	0.37* [0.04–0.63]	0.75*** [0.55–0.87]
Cyanuric Acid (mg/L)	0.38** [0.05–0.64]	0.60*** [0.33–0.78]

*p ≤ 0.05; **p ≤ 0.01; ***p ≤ 0.001

The validation of the proposed methodology demonstrated acceptable agreement with standard laboratory measurements across various water quality parameters. Correlation coefficients for key indicators, including pH, lead, total hardness, and iron, exceeded 0.85, suggesting that the method is reliable for field-based water quality assessments. Parameters such as total chlorine and free chlorine showed the highest correlation with laboratory results, indicating strong performance for these indicators. Other parameters, such as cyanuric acid and nitrate, exhibited moderate correlations, suggesting that while the method is sufficient for trend detection, it may require additional refinement for highly precise measurements. This is consistent with the inherent variability of test strips for certain analytes and the challenges posed by their chemical sensitivities.

Overall, the correlations were moderately strong, indicating that the improved methodology likely provided meaningful enhancements. Additionally, given the strength of the observed correlations and our sample size (*n* = 34), low *p*-values (< 0.001) are somewhat expected and reflect strong statistical evidence against the null hypothesis (no correlation). Certain parameters, such as Total and Free Chlorine, showed high correlations, possibly due to their absence in the water leading to all assessments showing either very low or 0 values. A similar pattern may apply to other parameters, such as heavy metals, which are less reactive with the test strips at lower concentrations (Sheets, 1998). That being said, the human–machine method was able to outperform the visual method on each parameter, demonstrating its effectiveness.

### Bland–Altman plots

A Bland–Altman analysis was conducted to evaluate the agreement between the proposed colorimetric test strip method and standard laboratory techniques across a range of water quality parameters.

**Fig. 4:**
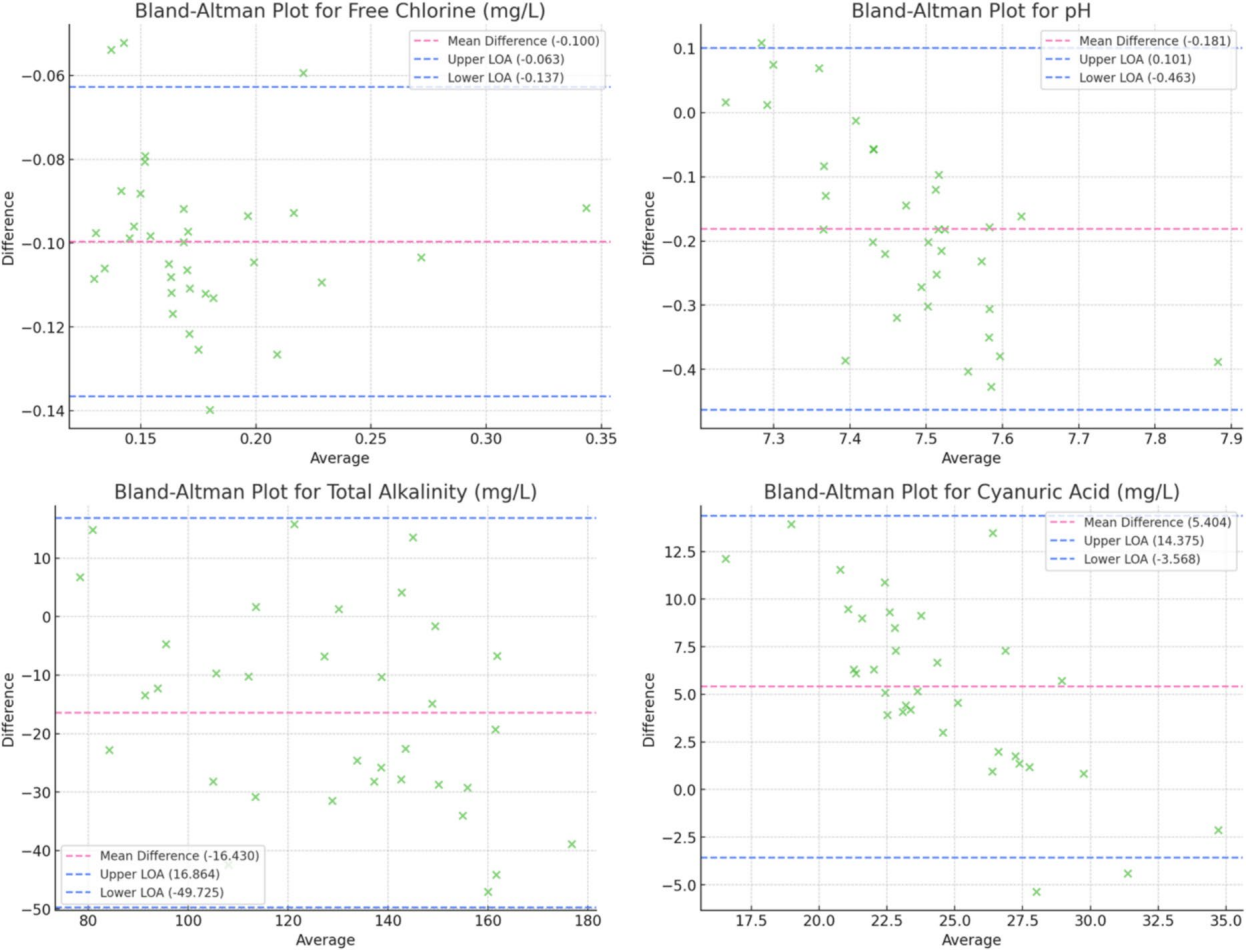
Bland–Altman plots for Free Chlorine, pH, Total Alkalinity, and Cyanuric Acid

For free chlorine (Fig. 4), a slight underestimation was observed, with a mean difference of + 0.1 mg/L. The broad limits of agreement highlight variability in low-concentration scenarios, likely influenced by ambient factors. Despite this variability, the method demonstrated reliability for general monitoring. The test strips consistently underestimated hardness, with a mean difference of − 15 mg/L and wide limits of agreement. This finding underscores challenges in accurately detecting hardness in high-mineral environments, suggesting that additional validation is necessary for precise applications.

pH (Fig. 4) had minimal bias (mean difference near 0) and narrow limits of agreement suggest strong performance for pH measurements; however, validation in extreme pH conditions is recommended to ensure accuracy across the full range. When considering total alkalinity and cyanuric acid (Fig. 4), alkalinity measurements showed a minor overestimation (+ 10 mg/L mean difference), while cyanuric acid was consistently underestimated (− 5 mg/L). Both parameters exhibited moderate variability, reflecting the limitations of test strip sensitivity at certain concentration ranges.

**Fig. 5:**
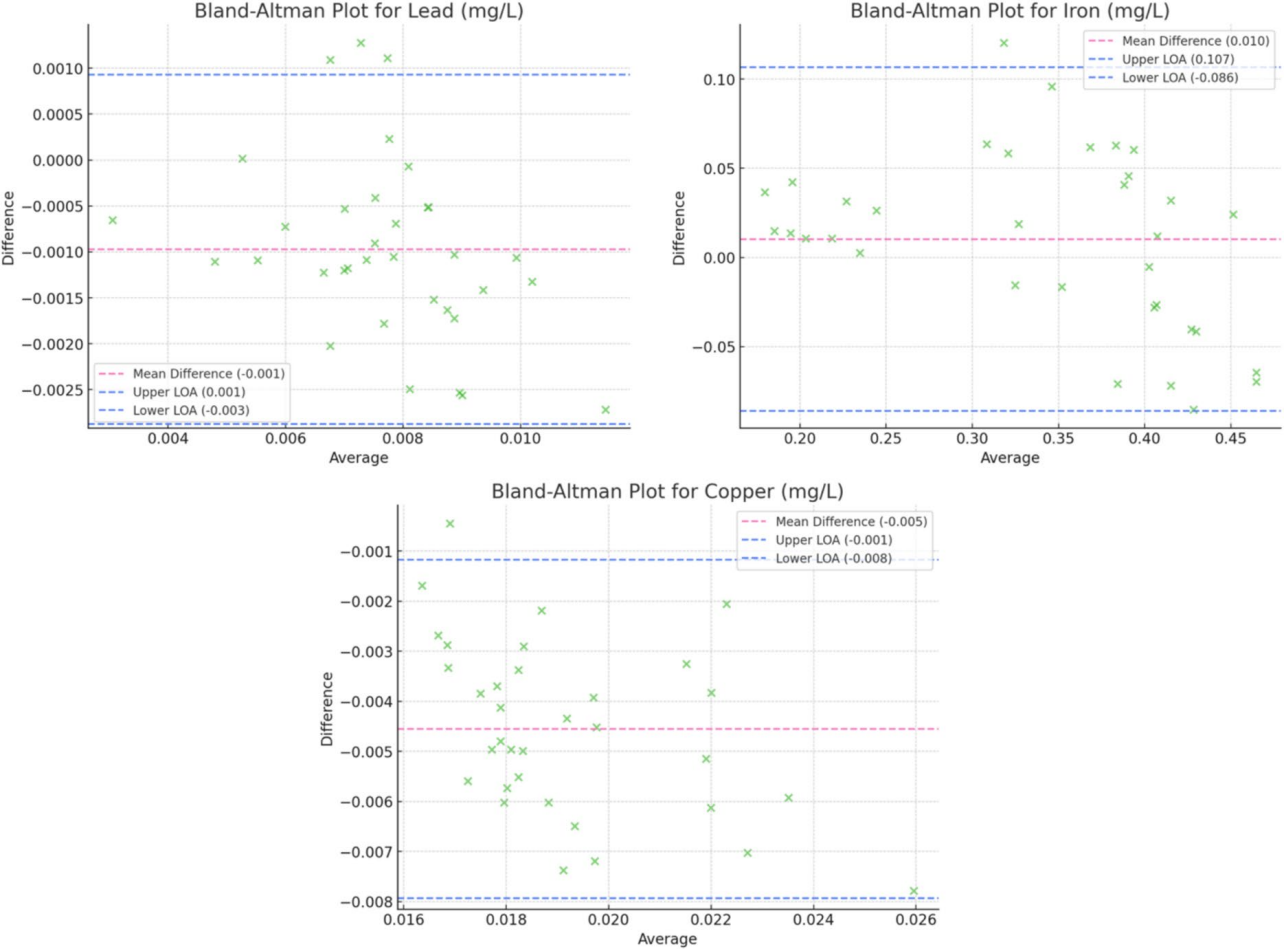
Bland–Altman plots for lead, iron, and copper

For heavy metals (lead, iron, and copper), lead and copper measurements exhibited slight overestimations (+ 0.001 mg/L and + 0.005 mg/L mean differences, respectively), while iron concentrations showed a slight underestimation (− 0.02 mg/L). Variability in these measurements emphasizes the general, though not highly precise, detection capabilities of test strips for trace elements. These results were not surprising as previous literature has found test strips struggle to adequately detect heavy metals in low concentrations (Sheets, 1998). The Bland–Altman plots for the heavy metals are shown below in Fig. 5.

**Fig. 6:**
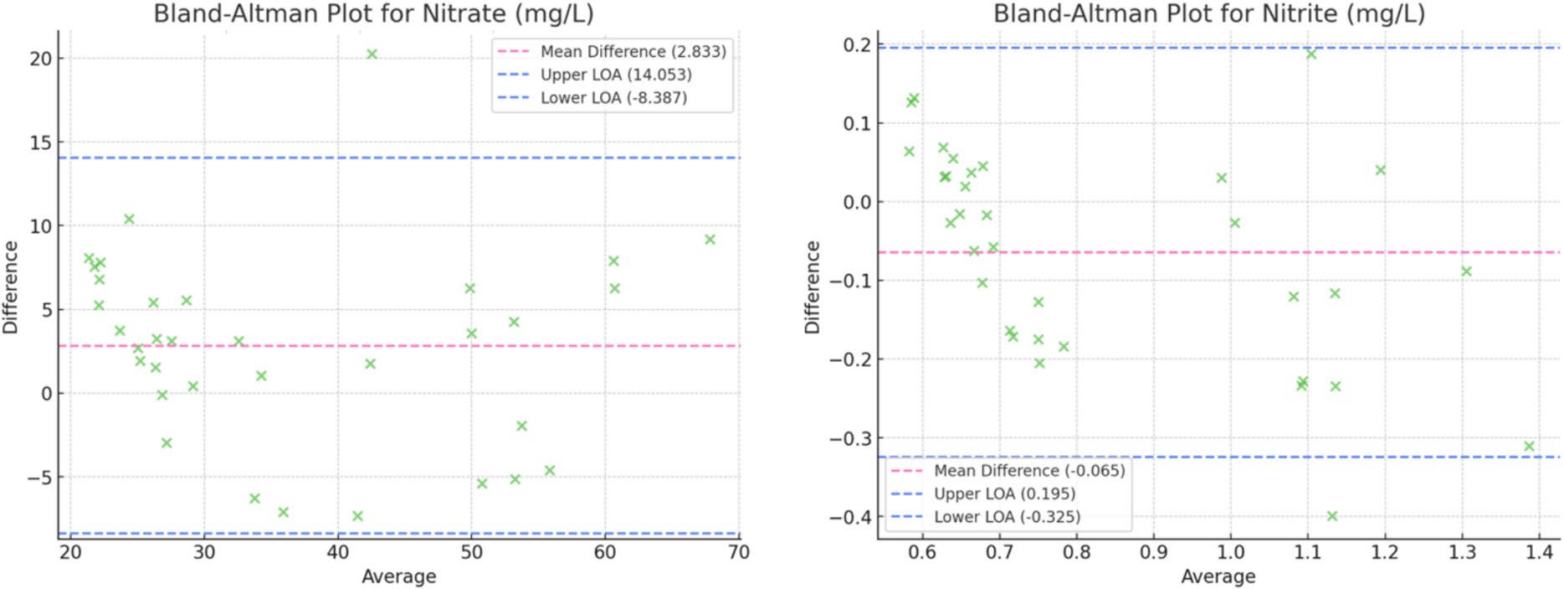
Bland–Altman plots for nitrate and nitrite

Lasty, for nutrients (nitrate and nitrite), underestimation of nitrate concentrations (− 3 mg/L mean difference) and slight overestimation of nitrite concentrations (+ 0.02 mg/L) were observed. While the method effectively captured the overall trends, similarly to heavy metals, its precision for these parameters may be limited at lower concentrations. The Bland–Altman plots for these are shown in Fig. 6 below.

Overall, parameters such as pH and free chlorine demonstrated narrow limits of agreement, indicating high consistency and suitability for field-based monitoring. Conversely, broader limits of agreement observed for parameters like nitrate and total hardness highlight the challenges of using test strips for precise quantification in more complex environmental conditions. However, they are able to find trends in the stream’s quality which could make them a useful tool for preliminary analyses. The analysis revealed no evidence of proportional bias, suggesting that the performance of the test strips is consistent across the range of concentrations evaluated. This finding supports the utility of the proposed method as a reliable and scalable approach for water quality monitoring in resource-limited or field settings.

### Performance analysis

The methodology’s ability to reliably measure pH, chlorine levels, heavy metals, and nutrient concentrations highlight its potential for diverse environmental monitoring applications. The integration of inverse distance weighting (IDW) further improved the precision of concentration estimates, particularly for test strip colors that did not precisely match any reference standard. This capability is particularly important for detecting subtle variations in water quality parameters, which are often critical for understanding pollution dynamics.

### Application to Seunggi Stream

The application of the methodology to Seunggi Stream provided insights into the spatial trends of water quality along the 7-km study area. Free chlorine levels varied significantly, with concentrations peaking at midstream urban centers, likely due to treated wastewater discharges and industrial effluents. Downstream fluctuations in free chlorine suggest natural dilution and degradation processes. Similarly, total hardness displayed substantial variability, with elevated levels observed in industrial and agricultural zones, indicative of mineral leaching and runoff from these areas.

Nutrient levels, including nitrate and nitrite, were elevated near agricultural zones, reflecting the influence of fertilizer runoff. Heavy metals such as lead, iron, copper, and zinc showed localized increases near industrial areas, consistent with known sources of contamination. The observed spatial trends demonstrate the methodology’s capability to capture site-specific variations, enabling targeted interventions for water quality management.

**Fig. 7:**
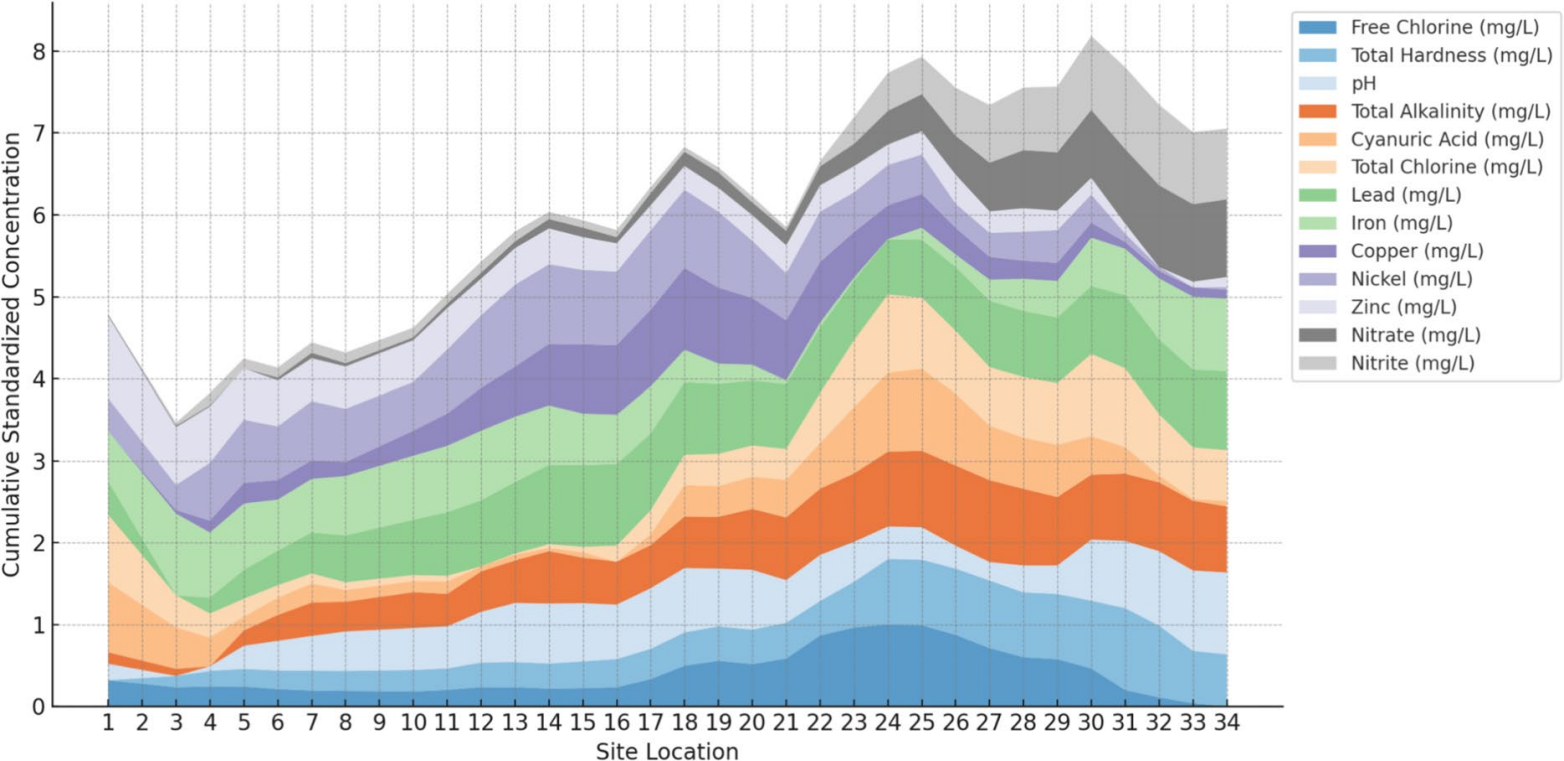
Cumulative concentration of pollutants in the stream (standardized)

Figure 7 above represents the cumulative concentrations as would be shown in the stream starting at the first sampling location (Site location = 1) and finishing at the end (Site location = 34). The figure standardizes the parameters with a min–max scaler to better facilitate comparison.

### Discussion of method advantages

The proposed methodology effectively addresses key limitations of traditional colorimetric water quality testing. By converting colors into quantifiable RGB values through a human–machine color sampling approach, it ensures accurate matching between test strips and reference charts. This process, combined with the application of objective mathematical algorithms, reduces the influence of human perception biases, and mitigates environmental inconsistencies such as lighting conditions, camera quality, and other external factors. The ability to extract and standardize colors directly from images using RGB conversion enables reliable comparisons between test samples and reference standards. This approach is particularly advantageous in field settings, where variability in external conditions can otherwise compromise data accuracy, or in places not necessarily equipped for research such as a classroom or room in a citizen’s house.

The interpolation technique employed in this study further enhances the precision of concentration estimates, improving on the accuracy of traditional visual matching methods. This advancement is valuable in environmental monitoring, where detecting subtle variations in water quality parameters is essential for timely intervention and effective mitigation efforts. The summary table below (Table 2) provides a comparison of the traditional methods (test strips), lab-based methods, and the proposed human–machine method across a range of criterion, including precision, subjective bias, reproducibility, robustness, deployment feasibility, cost-effectiveness, and speed of results.

**Table 2:** Summary table of different methods

Criterion	Traditional visual	Lab-based methods	Human–machine method (ours)
Precision	✗	✓✓	✓
Subjective Bias	✗	✓✓	✓✓
Reproducibility	✗	✓✓	✓
Robustness	✗	✓	✓
Deployment Feasibility	✓✓	✗	✓✓
Cost-Effectiveness	✓✓	✗	✓✓
Speed of Results	✓	✓✓	✓✓

Legend:* ✗* = poor performance,* ✓* = average performance,* ✓✓* = high performance

While traditional lab-based testing offers high precision and accuracy, they come with significant drawbacks, including high costs, the need for specialized equipment, and the requirement for trained personnel. These factors can limit their deployment, especially in resource-limited settings or for widespread environmental monitoring.

The proposed human–machine method addresses these limitations by utilizing accessible technology, such as smartphones, to perform colorimetric analyses. This approach offers several key advantages through the following: Eliminating the need for expensive reagents and specialized equipment, the method reduces overall costs, making it more feasible for widespread use; The use of portable and widely available devices allows for more frequent and extensive testing across various locations, facilitating comprehensive environmental monitoring; The user-friendly design of the system minimizes the requirement for extensive training, enabling a broader range of individuals to conduct accurate analyses.

By capitalizing on these advantages, the proposed human–machine method enhances the practicality and accessibility of colorimetric analysis, making it a valuable tool for applications where traditional lab-based methods may not be feasible.

## Practical implications

This methodology offers a cost-effective and accessible solution for water quality monitoring, especially in regions with limited access to laboratory facilities. Its reliance on readily available technologies, such as smartphones and web applications, makes it suitable for community-based monitoring programs and resource-limited settings. By providing accurate and objective assessments of water quality, the method supports timely decision-making and intervention, contributing to the protection of aquatic ecosystems and public health.

A preliminary cost comparison indicates savings using our hybrid approach. Traditional laboratory methods typically involve initial investments exceeding $5000–$10,000 USD (e.g., spectrophotometers, titration setups, chromatography systems), with additional recurring costs for reagents and calibration. In contrast, our method primarily leverages consumer-grade smartphones ($100–$1000 USD) and widely available colorimetric test strips (approximately $0.20–$1.00 USD per test), providing substantial accessibility and affordability advantages. Additionally, the proposed method requires no specialized laboratory facilities or personnel to conduct the water quality assessments.

While the method does not aim to replace laboratory testing, it complements traditional approaches by offering rapid, field-deployable assessments. The ability to automate color analysis and reduce subjectivity streamlines the monitoring process, making it an efficient tool for environmental professionals, researchers, and policymakers.

## Policy implications

The adoption of the proposed methodology has implications for enhancing water quality management frameworks. Policymakers can integrate RGB-based colorimetric analysis into regional and national water monitoring programs to improve data collection, especially in resource-constrained settings. The low-cost and accessible nature of this technology makes it an ideal candidate for decentralized monitoring efforts, empowering local governments and community organizations to take an active role in water quality assessment and management.

Incorporating this approach into policy planning can also facilitate more responsive and adaptive water governance. For example, the real-time data generated by this method can inform decision-making processes, enabling the rapid identification of contamination events and the implementation of mitigation strategies. Standardizing the use of RGB-based analysis in regulatory frameworks could ensure consistency in monitoring practices and strengthen compliance with water quality standards.

## Limitations and future work

While the proposed methodology offers significant advantages, several limitations should be acknowledged and addressed in future research. Notably, moderate correlations observed for analytes such as cyanuric acid and nitrate indicate areas where accuracy can be further improved. Refinement of the RGB-to-concentration mapping process, possibly through more advanced calibration routines or expanded reference datasets, could enhance detection precision for these parameters.

The study was conducted within a relatively uniform environmental setting limiting the scope of validation. Although results were encouraging, broader testing across water sources with varying characteristics, such as turbidity, organic matter content, and contaminant profiles, would be beneficial to further evaluate the method’s robustness and generalizability. As currently, the approach does not account for turbidity or background color interference, both of which can influence test strip color development. Future work could explore mitigation strategies, including repeated measurements, pre-filtration steps, or normalization techniques to minimize the impact of such confounding variables.

Seasonal and longitudinal studies are also recommended to assess the method’s stability under varying climatic and environmental conditions. Additionally, inter-laboratory comparisons would aid in evaluating reproducibility and support efforts to standardize the methodology for wider adoption. Although manufacturing-related variations in test strip coloration appeared minimal in this study, such inconsistencies could affect measurement reliability. Incorporating multiple test strips per sample and averaging the results may help reduce variability, though improved quality control from manufacturers remains essential.

Beyond methodological refinement, future research could explore integration of this approach into broader water monitoring frameworks. Emphasis should be placed on calibration for diverse environmental contexts, as well as compatibility with existing monitoring systems. Such efforts will be critical for positioning this hybrid human–machine methodology as a practical, standardized tool in environmental management and citizen science initiatives.

## Conclusion

The results of this study demonstrate the effectiveness of the proposed RGB-based colorimetric analysis for water quality monitoring. By addressing the limitations of traditional visual assessments and leveraging objective computational techniques, this method offers a reliable, scalable, and cost-effective alternative for field-based water quality assessments. Its ability to detect spatial trends and refine concentration estimates enhances its utility for environmental monitoring and public health protection.

From a policy perspective, integrating this technology into water management frameworks could improve data collection and compliance monitoring, particularly in resource-limited settings. Its affordability and accessibility support decentralized and community-driven efforts, empowering local stakeholders to actively participate in water quality management. Standardizing this method within regulatory frameworks could ensure consistent monitoring practices and foster rapid responses to contamination events.

By bridging scientific innovation with grassroots action, this approach has the potential to democratize water quality monitoring, strengthen public awareness, and enhance collaboration across sectors. Future refinement and policy incentives could further solidify its role in sustainable water governance.

## Data Availability

The data supporting the findings of this study are available upon request.
